# Biochemical mechanisms and molecular interactions of vitamins in cancer therapy

**DOI:** 10.1016/j.cpt.2024.05.001

**Published:** 2024-05-16

**Authors:** Abdullahi T. Aborode, Isreal A. Onifade, Mercy M. Olorunshola, Gladys O. Adenikinju, Ibude J. Aruorivwooghene, Adeboboye C. Femi, Osasere Jude-Kelly Osayawe, Abraham Osinuga, Ebenezer A. Omojowolo, Adekunle F. Adeoye, Segun Olapade, Ibrahim O. Adelakun, Ogundepo D. Moyinoluwa, Oluwatosin M. Adeyemo, Godfred Y. Scott, Ruth A. Ogbonna, Emmanuel A. Fajemisin, Omama Ehtasham, Soyemi Toluwalashe, Adetolase A. Bakre, Ridwan O. Adesola, Seto C. Ogunleye, Nnenna R. Anyanwu, Terungwa H. Iorkula

**Affiliations:** a Department of Chemistry, Mississippi State University, Starkville, MS 39759, USA; b Department of Biology, University at Albany, Albany, NY 12222, USA; c Department of Biological Sciences, State University of New York at Binghamton, Binghamton, NY 13902, USA; d Department of Biological and Environmental Sciences, University of Rhode Island, Kingston, RI 02881, USA; e Department of Biological Sciences, University of Kansas Medical Center, Kansas, KS 66103, USA; f Department of Microbiology, Federal University of Technology, Akure 340110, Nigeria; g Department of Chemistry and Biochemistry, Brigham Young University, Provo, UT 84602, USA; h Department of Chemical and Biomolecular Engineering, University of Nebraska-Lincoln, Lincoln, NE 68588, USA; i Department of Chemistry, University of Albany, State University of New York, Albany, NY 12222, USA; j Department of Mathematics and Statistics, Georgia State University, Atlanta, GA 30302, USA; k Department of Chemistry, University of Louisville, Louisville, KY 40208, USA; l Department of Biochemistry, Federal University of Technology, Akure 340110, Nigeria; m Department of Medical Diagnostics, Kwame Nkrumah University of Science and Technology, Kumasi AK385, Ghana; n Department of Research and Development, Nasarawa State AIDS and STI Control Program, Nasarawa, Lafia 962101, Nigeria; o Department of Integrative Biomedical Science, University of Cape Town, Cape Town 7701, South Africa; p Department of Medicine and Surgery, Karachi Medical and Dental College, Karachi 74700, Pakistan; q Department of Medicine, Lagos State University College of Medicine, Lagos 10010, Nigeria; r Department of Veterinary Medicine, Faculty of Veterinary Medicine, University of Ibadan, Ibadan 200005, Nigeria; s Faculty of Pharmaceutical Sciences, University of Jos, Plateau, Jos 930003, Nigeria

**Keywords:** Vitamins, Cancer, Biochemical, Molecular interactions, Therapy

## Abstract

Recently, the potential role of vitamins in cancer therapy has attracted considerable research attention. However, the reported findings are inconsistent, with limited information on the biochemical and molecular interactions of different vitamins in various cancer cells. Importantly, the presence of vitamin receptors in tumor cells suggests that vitamins play a significant role in the molecular and biochemical interactions in cancers. Additionally, studies on the efficacy of vitamin supplementation and dosage levels on tumor progression and mortality risk have yielded inconsistent results. Notably, molecular and biochemical investigations have reported the function of vitamins in the proliferation, growth, and invasiveness of tumor cells, as well as in cell cycle arrest and inflammatory signaling. Additionally, different vitamins may regulate the cancer microenvironment by activating various molecular pathways. Vitamins significantly affect immunological function, antioxidant defense, inflammation, and epigenetic control, and can improve treatment outcomes by affecting cell behavior and combating stress and DNA damage. However, further research is necessary to confirm the efficacy of vitamins, establish ideal dosages, and develop effective cancer prevention and treatment plans. Individualized supplementation plans guided by medical knowledge are crucial to achieving optimal results in clinical and preclinical settings. In this review, we critically evaluated the effects of different vitamins on the risk and development of cancer. Additionally, we examined the potential of vitamin supplements to enhance the efficacy of drug therapy and counteract resistance mechanisms that often arise during cancer treatment.

## Highlights

• Chronic inflammation increases the risk of cancer.
• Vitamin supplementation may lower the risk of cancer in humans.
• Vitamins C and E protect cells from cancer-causing oxidative damage, DNA repair, cell growth, and division depend on vitamins such as vitamin B.

## Graphical abstract

**Figure:**
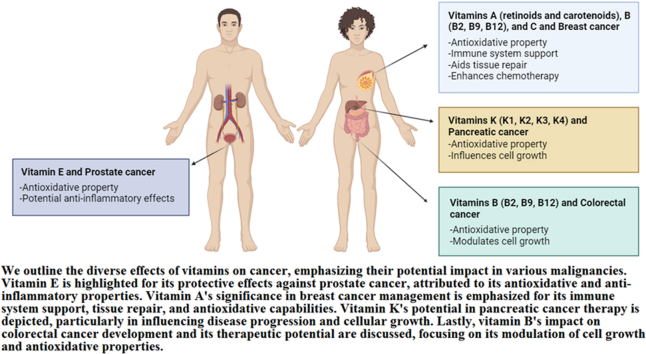

## Introduction

Vitamin D (Vit D) deficiency has been linked to the risk and development of solid and non-solid cancers.^1^^,^^2^ Vit D interferes with cellular iron homeostasis, causing oxidative stress and cell death in cancer cells, and its deficiency is linked to an increased risk of breast cancer.^3^ Additionally, Vit D receptor (VDR) is upregulated in tumor tissues, enhancing its antineoplastic effects.^1^ Moreover, Vit D treatment reduces glioblastoma cells movement and invasive phenotypes. Notably, Vit D may also improve anti-tumor activity by upregulating VDRs, thus producing similar inhibitory effects *in vivo* without causing side effects, such as hypercalcemia.^4^

Research findings indicate that Vit D, when used in combination therapy, improves the efficacy of anti-cancer drugs, such as cisplatin/gemcitabine^5^, ^6^, ^7^ and proton therapy,^5^ in various cancer models. Although studies have been performed on the effect of Vit D on cancer outcomes, there is no universally accepted dose or treatment duration in cancer therapy. Moreover, ongoing research has produced varying conclusions based on the type and stage of cancer and specific patient characteristics.^6^^,^^7^ Vit D supplementation improves specific aspects of cancer treatment, such as lowering the risk of recurrence or enhancing survival rates.^8^ However, the precise amount and duration of supplementation differ considerably among studies.^8^, ^9^, ^10^, ^11^, ^12^

A 2019 study by JAMA Oncology on the impact of high-dose (4000 international unit [IU]/day) and standard-dose (400 IU/day) Vit D supplementation in patients with metastatic colorectal cancer revealed that patients in the high-dose group had better progression-free survival outcomes than those in the standard-dose group.^13^ Although patients with cancer commonly have below-average Vit D levels, the extent of this deficit may differ among patients. Importantly, the increase in Vit D levels following supplementation may vary based on factors such as supplementation dosage, initial levels, and individual patient reactions.^13^ Accordingly, the optimal dosage and duration of supplementation may differ based on individual patient characteristics and should be customized to meet the unique requirements of each patient. Epidemiological studies have examined the association between Vit D levels and cancer risk.^14^^,^^15^ Notably, the risk of specific types of cancers is lower in individuals with optimal Vit D levels.^15^^,^^16^

Vitamin C (Vit C) is well known for its potent antioxidant activity and is naturally found in several fruits and vegetables. Notably, the role of Vit C in cancer metastasis suggests that it may possess anti-cancer properties.^17^^,^^18^ Several studies have demonstrated the ability of pharmacological doses of Vit C, either alone or in combination with clinically used drugs, to exert beneficial effects in various human cancer models.^17^, ^18^, ^19^ Similarly, dehydroascorbate, an oxidized form of Vit C, causes energy crisis and cell death in cancer cells.^20^

Furthermore, chemotherapy may be improved by using Vit C as an adjuvant,^21^ and Vit C administration also reduces the side effects of chemotherapy.^22^ Although the results of studies on the effect of Vit C on prostate cancer are inconclusive, Lee et al.^23^ reported that dietary Vit C intake is promising for preventing and treating prostate cancer.

Serum vitamin B (Vit B) levels are associated with different cancers.^24^ Moderate doses of Vits B2, B9 (folic acid), B6, and B12 can reduce the risk of colorectal cancer depending on the stage of cancer growth, as moderate doses of Vit B are more effective in enhancing survival rates than high doses.^24^^,^^25^ Vits B2, B6, and B9 can inhibit the growth of monocytic lymphoma and have anti-tumor properties,^26^ whereas folic acid and Vit B12 supplementation have been shown to protect against breast cancer.^26^^,^^27^ Additionally, folic acid in the plasma can inhibit breast cancer cell proliferation and metastasis, lowering the risk of recurrence and metastasis.^28^ Moreover, the Vit B6 Schiff base Mn (II) complex is a potent anti-tumor agent used to treat breast cancer.^29^ However, other findings have linked Vit B to an increased risk of breast cancer.^30^, ^31^, ^32^

Vitamin E (Vit E) is a dietary antioxidant that exists in different natural forms, including (RRR)-α-tocopherol (αT), β-tocopherol (βT), γ-tocopherol (γT), and δ-tocopherol (δT) and (R)-α-tocotrienol (αTE), β-tocotrienol (βTE), γ-tocotrienol (γTE), and δ-tocotrienol (δTE). Notably, γT, δT, γTE, and δTE have been shown to suppress tumor development in relevant animal cancer models, whereas αT was frequently ineffective in similar preclinical study.^33^ Similarly, some studies, including animal models, have shown that γT has greater anti-inflammatory and anti-cancer properties than αT.^34^^,^^35^ Several epidemiological and observational studies have investigated the association between Vit E and cancer risk.^36^, ^37^, ^38^ Considering that different forms of Vit E have anti-cancer potential, preclinical research is needed to validate and improve their efficacy in cancer prevention and treatment.

Vitamin A (Vit A) is comprised of retinol, its derivatives (retinoids), and carotenoids (provitamin A), which can be converted into retinol and retinoids. Vit A and carotenoids protect against photo energy, boost the immune system, and modulate oxidative stress, thereby regulating cancer cell growth and differentiation.^39^ Research findings indicate that Vit A and its derivatives suppress the growth and differentiation of cancer cells.^39^^,^^40^ For instance, serum retinol or β-carotene levels are significantly lower in people with breast cancer.^41^ Additionally, serum concentrations of Vit A and carotenoids are lower in patients with advanced stages of breast cancer.^41^, ^42^, ^43^

β-carotene supplements and their possible effects on lung cancer risk have long been topics of interest.^43^, ^44^, ^45^ β-carotene is a natural precursor of Vit A found in various fruits and vegetables. Research indicates that consuming β-carotene supplements, especially in high amounts, could have negative effects on specific groups such as smokers and those susceptible to lung cancer.^45^ The Beta-Carotene and Retinol Efficacy Trial (CARET), conducted in the 1990s, examined the impact of β-carotene and retinol supplements in lung cancer prevention in high-risk individuals, such as smokers and asbestos-exposed workers, and confirmed the negative effects of Vit A supplements in high-risk individuals.^46^ Additionally, the CARET trial indicated a higher incidence of lung cancer among individuals who took β-carotene supplements, especially smokers.^46^ This surprising discovery raised concerns over the safety of β-carotene supplementation, particularly in populations at high risk of lung cancer. Various explanations have been suggested for the observed increase in lung cancer risk associated with β-carotene supplementation. Although it is important to recognize that the CARET study and other studies have highlighted the potential risks associated with β-carotene supplements and lung cancer, these findings may not be universally relevant to all groups or situations. Further research is necessary to elucidate the mechanisms underlying the reported effects and ascertain whether specific subgroups are more susceptible to the negative effects of β-carotene supplementation.

**Figure 1:**
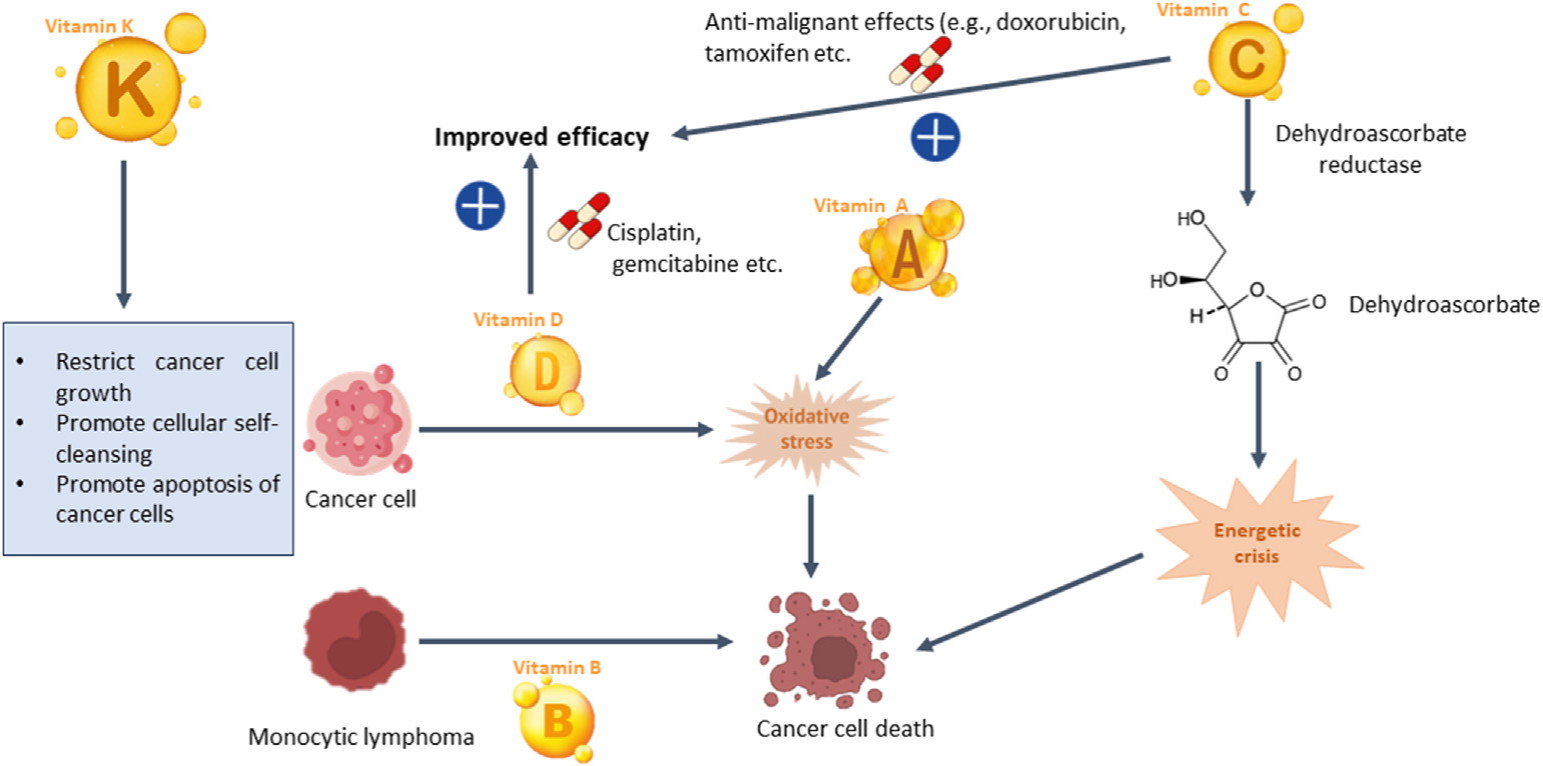
Overview of vitamins targeting various cancers.

Vitamin K (Vit K) is a group of organic compounds that naturally occurs in food and is either produced by bacteria, synthesized within the body, or obtained through synthetic means,^47^ as shown in Figure 1. This group comprises Vits K1 (phylloquinone), K2 (menaquinones), K3 (menadione), and K4 (menadione sodium phosphate), all of which share the common structure of a 2-methyl-1,4-naphthoquinone ring with variations in the C-3 side chains.^48^ Phylloquinone and menaquinone are natural forms of Vit K found in the diet. Major sources of Vit K1 include green leafy vegetables and vegetable oils, whereas menaquinone is predominantly present in animal foods, particularly the liver, as well as fermented foods.^48^^,^^49^ They are lipid-soluble,^48^ apart from the ones of synthetic origin, which are water-soluble.^48^ Vit K performs several important functions in the body. For example, Vit K acts as a co-factor for gamma-glutamyl carboxylase, which is responsible for the post-translational carboxylation of glutamic acid into gamma-carboxyglutamic acid.^49^ Notably, the involvement of Vit K in prothrombin production contributes to blood clotting. Additionally, Vit K plays a vital role in bone metabolism and the vascular system, among other crucial functions.^50^

Regarding its association with cancer, the role of Vit K remains an area of limited comprehensive research and the precise mechanisms underlying the anti-cancer effects of Vit K remain unclear. However, some observed effects of Vit K include restricting cell growth, encouraging cellular maturation, restricting the spread of cancer, and promoting cellular self-cleansing or programmed cell death.^49^^,^^50^ Notably, the specific anticancer effects may vary depending on the cancer type, Vit K form, and experimental design.^51^ Existing literature suggests that Vit K may possess both preventive/restrictive (anti-cancer) and supportive properties in cancer therapy.^50^^,^^51^ Yu et al.^49^ reported that there is a nonlinear relationship between dietary Vit K intake and the risk of pancreatic cancer, highlighting the potential preventive role of Vit K against this particular cancer type.

Cancer is one of the biggest health issues worldwide, with increasing prevalence and socio-economic impact. Despite breakthroughs in cancer treatment, researchers have continued to investigate novel therapeutic strategies, including vitamin therapy. Epidemiological research suggests that vitamins may prevent cancer, improve therapy efficacy, and facilitate recovery.^52^^,^^53^ For example, Vit D levels are inversely associated with the risk of colorectal, breast, prostate, and pancreatic cancers.^53^^,^^54^ Additionally, observational studies suggest that increasing Vit D levels may enhance cancer survival.^55^^,^^56^ Importantly, Vit D may inhibit cancer progression by regulating cell proliferation, apoptosis, angiogenesis, and immunological functions. However, further studies, including randomized controlled trials, are required to determine the molecular mechanisms and efficacy of Vit D supplementation in cancer treatment.

Vit C has been extensively studied in cancer treatment owing to its antioxidant properties.^57^ Epidemiological studies on the association between Vit C levels and cancer risk have reported varying results, with some studies indicating preventive benefits against certain malignancies.^57^^,^^58^ For example, preclinical and clinical trials have reported encouraging outcomes following intravenous (IV) administration of high-dose of Vit C as a cancer adjuvant.^58^^,^^59^ Vit C may block angiogenesis, induce oxidative stress, and modulate immunological functions in cancer. However, Vit C supplementation in cancer therapy requires further research on dosing, timing, and patient selection.

Vit E, a fat-soluble antioxidant, may also be effective against cancers. A previous study identified a slight protective effect of Vit E intake against prostate cancer, whereas other studies found no meaningful connection.^60^ Notably, the antioxidant, cell signaling pathway modification, and immunomodulatory effects of Vit E may contribute to inhibit cancer development and progression.^60^ Further research is necessary to determine the role and mechanisms of Vit E in cancer treatment and identify subgroups that may benefit from Vit E supplementation. Extensive studies on Vits D, C, and E have suggested their cancer prevention and therapeutic benefits.^61^ However, further studies, including randomized controlled trials, are needed to confirm these findings, elucidate the underlying mechanisms, and determine appropriate vitamin supplement dosing and timing for cancer treatment.

A clinical study investigated the effect of cholecalciferol on the survival of individuals newly diagnosed with cancer and those with low Vit D levels.^62^ Vit D supplementation may enhance tumor response and survival rates, and prolong the time before treatment in patients with Vit D deficiency. Clinical research investigating the use of multivitamin supplements for cancer prevention has shown inconclusive outcomes.^62^^,^^63^ A collaborative trial between Finland and the National Cancer Institute (NCI), known as the Alpha-Tocopherol, Beta-Carotene Cancer Prevention Study (ATBC), involving 29,133 male smokers aged 50–69 years, did not show a decrease in lung cancer as expected prior to the trial.^63^ Participants were randomly allocated to one of four supplementation regimens, which included αT (50 mg) and β-carotene (20 mg) either together, separately, or a placebo. Notably, observational studies have indicated that high consumption of calcium and Vit D is linked to a reduced risk of developing colorectal cancer and polyps.^64^ Vit D influenced the effect of calcium supplementation on the risk of adenomas,^64^ with calcium supplements reducing cancer risk only in individuals with a Vit D intake above the median. In this current review, we examined the potential of vitamin supplements to enhance the efficacy of drug therapy and counteract resistance mechanisms that often arise during cancer treatment.

## Biochemical mechanism of vitamins on cancer cells

The most outstanding feature of malignant tumor metabolism, especially that of rapidly growing tumors, is aerobic glycolysis, which enables cancer cells to maintain their energy levels and produce nucleotides, amino acids, and fatty acids to sustain their proliferation capacity.^65^^,^^66^ Vitamins and minerals are essential for aerobic respiration, the mechanism through which regular cells produce energy in the presence of oxygen.^66^ Vits B1 (thiamine), B2 (riboflavin), B3 (niacin), B5 (pantothenic acid), and B12 (cobalamin) act as coenzymes or coenzyme precursors at different stages of aerobic respiration. Importantly, they assist in converting glucose into pyruvate during glycolysis and have important functions in the citric acid cycle (Krebs cycle) and the electron transport chain. Vit C functions as an antioxidant by shielding cells from the harmful effects of reactive oxygen species (ROS) produced during aerobic respiration.

Iron is a constituent of hemoglobin, a protein found in red blood cells that is responsible for transporting oxygen from the lungs to tissues.^66^ Iron also acts as a cofactor for enzymes that participate in the electron transport chain. Magnesium acts as a cofactor for numerous enzymes that play a role in adenosine triphosphate (ATP) generation and is crucial for the activity of ATP synthase, the enzyme responsible for ATP synthesis. Zn serves as a cofactor for enzymes involved in different metabolic pathways, such as glycolysis and the citric acid cycle.^67^

**Figure 2:**
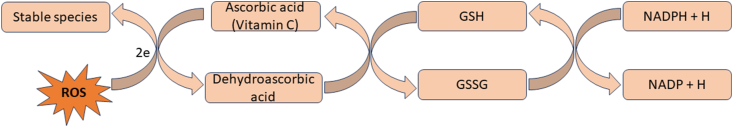
ROS interactions with vitamin C. GSH: Glutathione; GSSG: Glutathione disulfide; NADP: Nicotinamide adenine dinucleotide phosphate; NADPH: Nicotinamide adenine dinucleotide phosphate; ROS: Reactive oxygen species.

Vitamin and mineral deficiencies can hinder aerobic respiration and cellular metabolism, resulting in impaired cellular respiration and the onset of fermentation. Deficiencies in vitamins, iron, magnesium, and other important nutrients may hinder the action of the enzymes involved in aerobic respiration, resulting in low ATP generation and limiting the cell's capacity to fulfill its energy requirements.^67^ Insufficient amounts of antioxidants, such as Vit C, can result in elevated oxidative stress in cells. ROS produced during incomplete aerobic respiration can harm cellular structures, such as DNA, proteins, and lipids, leading to the potential disruption of cellular function and elevated risk of mutations^67^ [Figure 2].

Cells may undergo anaerobic fermentation to fulfill their energy requirements if they are unable to produce sufficient energy through aerobic respiration owing to nutrient deficits or other reasons. Fermentation is less effective than aerobic respiration in ATP production and can result in the accumulation of lactate and other waste products. Long-term dependence on fermentation can change cellular metabolism and support the survival and growth of cancer cells, which often favors anaerobic metabolism even when oxygen is available (known as the Warburg effect).^68^ Vitamins and minerals are essential for both aerobic respiration and cellular metabolism. Insufficient levels of these nutrients may hinder energy generation, elevate oxidative stress, and encourage the transition to fermentation, possibly leading to cellular harm and cancer development.^68^ Therefore, the inhibition of glycolysis and other pathways of energy provision for cancer cells can halt tumor growth, eventually leading to partial or complete remission. Importantly, vitamins with verified anti-cancer properties include Vits A, B1, C, D, and E.

Vit A exerts inhibitory effects on the Janus kinase signal transducer and activator of the transcription (JAK-STAT) pathway.^69^ Vit B1 (thiamine) administration enhances the absorption of thiamine specifically into tumor cells. Vit C facilitates the proliferation and development of T cells and natural killer (NK) cells, both of which are crucial components of the immune system against cancer.^70^ Vit D decreases the risk of cancer progression through its inhibitory effects on glucose metabolism. Moreover, Vit E can suppress cellular proliferation and trigger apoptotic cell death in several cancer cell lines.^70^

### Vitamin A

**Figure 3:**
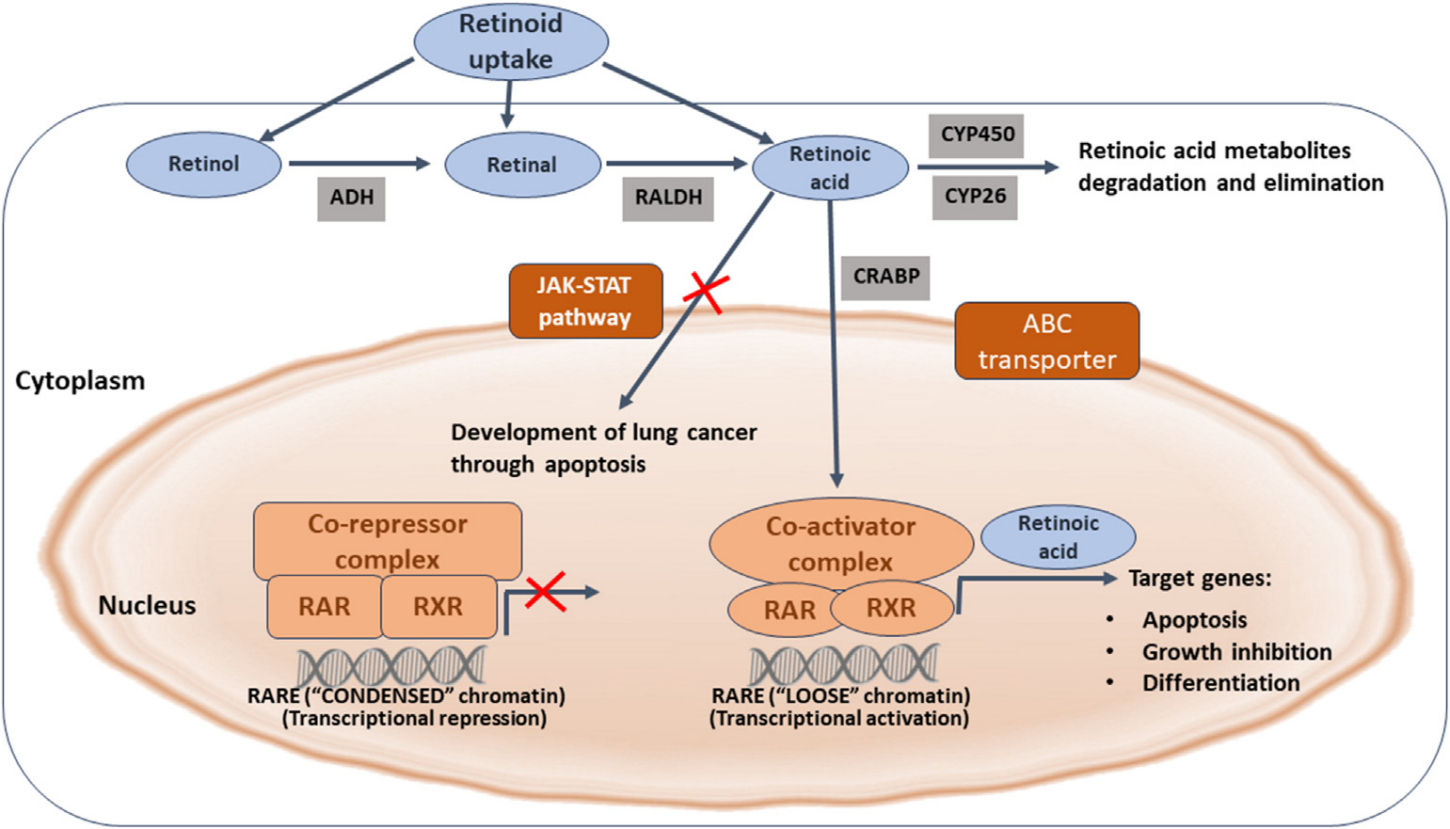
Summary of the role of vitamin A in the treatment of cancer. ABC: ATP-binding cassette; ADH: Antidiuretic hormone; CRABP: Cytosolic retinoic acid-binding protein; CYP26: Cytochrome P450 family 26; CYP450: Cytochrome P450; ER: Estrogen receptor; JAK: Janus kinase; RAR: Retinoic acid receptor; RALDH: Retinal dehydrogenase; RARE: Retinoic acid response element; Rb: Retinoblastoma; RXR: Retinoid X receptor; STAT: Signal transducer and activator of the transcription.

Retinoids can regulate various components of the skin's immune system, which influence the behavior of cutaneous T cell lymphoma cells and their surroundings. Additionally, retinoids directly affect malignant T lymphocytes [Figure 3]. Retinoic acid (RA) amide, a metabolite of Vit A, inhibits the JAK-STAT pathway, which plays a crucial role in preventing the development of lung cancer by facilitating the apoptosis of precancerous cells. Activation of the JAK-STAT signaling pathway occurs via the formation of a scaffold comprising retinol-binding protein 4 (RBP4) bound to its ligand and stimulated by RA 6 (STRA6).^39^

An *in vitro* study revealed that carotenoids can hinder the growth of tumor cells in both estrogen receptor (ER)-positive and ER-negative breast cancers.^71^ However, the mechanisms underlying this inhibition differ between the two types of breast cancer. Retinoids can inhibit cyclin D and telomerase levels in ER-positive tumors, leading to cell cycle arrest.^71^ Additionally, retinoids can upregulate the tumor proteins 53 (p53), cyclin-dependent kinase inhibitor 1 (p21), and retinoblastoma (Rb) protein, resulting in the inhibition of the growth of ER-negative tumors.^39^^,^^70^

Previous studies have examined the possible impact of RA on various breast cancer cell lines including Malondialdehyde (MDA), Michigan Cancer Foundation-7 (MCF-7), and demonstrated its inhibitory effects on proliferation.^72^^,^^73^ RA can inhibit the activity of antioxidant enzymes, such as peroxidase, catalase, and glutathione (GSH), in human breast cancer cells.^73^ Moreover, elevated levels of RA may result in the aggregation of molecules, leading to an increase in their size and impeding their transport across cellular membranes.^74^

Notably, the anti-cancer effects of retinoids in basal cell carcinoma (BCC) are mediated through the induction of apoptosis and modulation of signaling pathways that play crucial roles in BCC development.^75^ RA induces the activation of the mitochondrial pathway, leading to the cessation of cell differentiation and initiation of apoptosis. Apoptosis occurs as a result of mitochondrial malfunction, production of ROS, and subsequent release of cytochrome c and activation of the caspase 8/t-BID cell death pathway.

### Vitamin B1

A comparative *in vitro* investigation on the effects of different concentrations of thiamine on the breast cancer cell line MCF and the non-tumorigenic cell line MCF-10A showed that there was a significant decrease in MCF-7 cell proliferation following treatment with high doses of thiamine (1 and 2 mg/mL) for 24 h.^76^ Moreover, there was a correlation between the observed decrease in glycolysis and activation of the pyruvate dehydrogenase (PDH) complex.^75^ Under hypoxic conditions, the hypoxia-inducible factor (HIF)-1α promotes an increase in thiamine absorption in tumor cells.^76^ In a study involving seven cancer cell lines subjected to hypoxic conditions, there was an increase in the expression of thiamine pyrophosphate kinase-1 (TPK1), an enzyme responsible for the conversion of thiamine to thiamine pyrophosphate (TPP).^75^^,^^76^ Despite the observed increase in TPK1 expression, a concurrent decrease in intracellular TPP levels was observed.

### Vitamin C

Several mechanisms have been proposed to explain the involvement of Vit C in cancer treatment and prevention.^77^ These mechanisms include enhancing the immune system function, promoting collagen synthesis, impeding metastasis through the inhibition of specific enzymatic reactions, suppressing tumor-causing viruses, addressing Vit C deficiency commonly observed in cancer patients, facilitating wound healing following cancer surgery, enhancing sensitivity to chemotherapy, reducing chemotherapy-induced toxicity, and counteracting specific carcinogens.

Additionally, Vit C can improve tumor inclusion and enhance the effectiveness of chemotherapy.^78^ Furthermore, it is important to examine the implications and efficacy of administering high IV doses of Vit C in cancer therapy. *In vitro* experiments showed that pharmacological concentrations of Vit C ranging from 0.3 to 20 mmol/L eliminated cancerous cells.^78^ Notably, the Vit C concentrations examined above were above the typical physiological level, which is typically approximately 0.1 mmol/L. Additionally, the ability of Vit C to destroy tumors can be attributed to its pro-oxidant properties. High doses of Vit C promote the production of hydrogen peroxide (H_2_O_2_), which is believed to play a role in its antitumor effects.

Vit C undergoes oxidation upon entering the internal milieu of the body, resulting in dehydroascorbic acid (DHA) formation. Subsequently, DHA is efficiently transported by glucose transporter 1 (GLUT-1) in mutant Kirsten rat sarcoma viral oncogene homolog (KRAS) or v-Raf murine sarcoma viral oncogene homolog B (BRAF) cells, thereby competing with glucose.^79^ DHA undergoes a nicotinamide adenine dinucleotide phosphate (NADPH)-facilitated conversion process within the cellular environment and is reduced by GSH to yield ascorbate. Overall, this phenomenon leads to a decrease in antioxidant levels within the cytosol and an increase in ROS levels within the cell.

An increase in ROS levels leads to the inactivation of glyceraldehyde 3-phosphate dehydrogenase (GAPDH) by oxidizing a cysteine residue located in the active region of the enzyme. Notably, ROS plays a role in the activation of poly (adenosine diphosphate [ADP]-ribose) polymerase (PARP), resulting in the depletion of nicotinamide adenine dinucleotide (NAD)^+^, an essential cofactor of GAPDH. Consequently, this leads to a further decrease in GAPDH levels, contributing to a complex metabolic reconfiguration. GAPDH inhibition can lead to a state of diminished ATP synthesis, sometimes referred to as “energy crisis.”^80^ Moreover, the administration of large doses of Vit C induced significant oxidative stress in breast cancer cell lines, resulting in ROS generation and DNA damage. Additionally, this therapy resulted in a decrease in crucial intracellular cofactors, including NAD^+^.

Cancer cells with elevated ROS levels and immune cells exhibiting a reactive response within the cancer microenvironment can induce the secretion of interleukin-6 (IL-6).^80^ IL-6 plays a significant role in suppressing immune response in several types of malignancies, including pancreatic, colorectal, and melanoma, following anti-programmed death-ligand 1 (PD-L1) therapy.^80^ IL-6-induced activities can be inhibited by ascorbic acid through the enhancement of TET2 activity, thereby presenting a novel concept for cancer treatment.^81^

Vit C inhibits the development of a hypoxic microenvironment, a critical element in cancer metastasis, by impeding the activation of HIF-1. Notably, HIF is involved in several phases of metastasis, including the initiation of epithelial-to-mesenchymal transition (EMT) and the promotion of angiogenesis.^81^ Additionally, HIF plays a role in promoting the evasion of cancer cells from immunological attack by inhibiting macrophage-associated phagocytosis and suppressing NK cell-mediated anti-cancer response.

### Vitamin D

**Figure 4:**
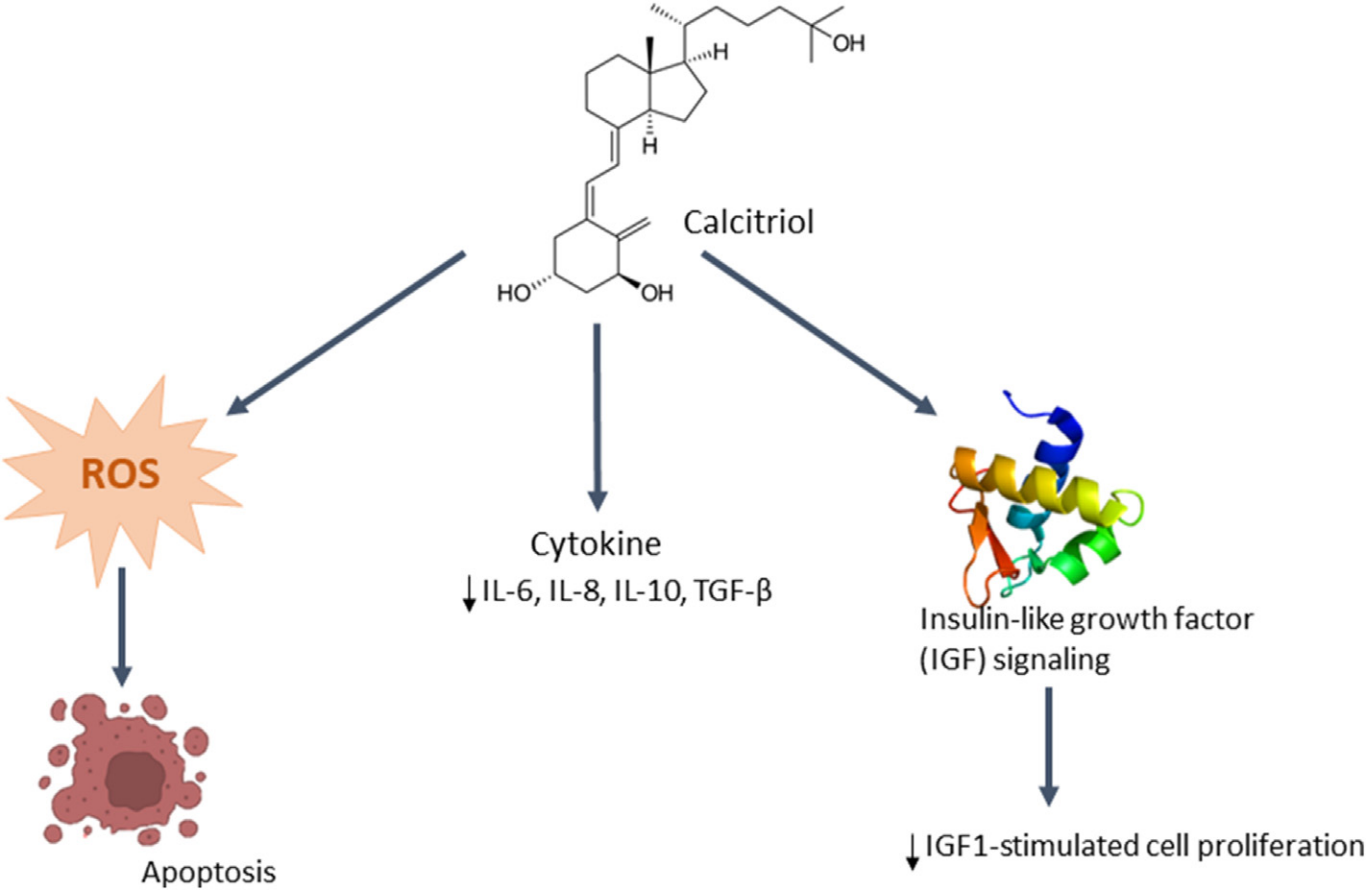
Summary of the role of vitamin D on cancer. IGF: Insulin-like growth factor; IL: Interleukin; ROS: Reactive oxygen species; TGF-β: Transforming growth factor beta.

The primary mechanism by which Vit D exerts its effects is through its hormonal form, known as 1,25-dihydroxy-vitamin D (1,25(OH)_2_D), as depicted in Figure 4. Several strategies have been employed to combat cancer cells, including impeding their development, promoting cellular differentiation, inducing programmed cell death (apoptosis) and self-degradation (autophagy), and hindering the formation of new blood vessels (angiogenesis) in metastatic tumors.^82^ Previous studies have provided empirical evidence that 1,25(OH)_2_D_3_ inhibits angiogenesis, tumor growth, and metastasis in the tumor microenvironment (TME).^82^^,^^83^

Another potential mechanism by which Vit D inhibits cancer progression is via the suppression or reversal of abnormal glucose metabolism, which includes the reduction of glucose uptake into cancerous cells.^83^ For example, 1,25(OH)_2_D treatment reduced GLUT1 messenger RNA (mRNA) and protein expression in prostate cancer cell models.^84^ Additionally, 1,25(OH)_2_D reduced GLUT1 mRNA and protein expression and decreased glucose absorption in the breast cancer cell lines MCF-7 and MDA-MB-231.^85^ Notably, the effect of 1,25(OH)_2_D on GLUT1 expression may vary depending on the specific stage or type of cancer. Similarly, a study on an *in vitro* model of early-stage breast cancer showed that 1,25(OH)_2_D treatment decreased glucose absorption but had no effect on GLUT1 mRNA levels.^85^ Apart from its role in glucose absorption, 1,25(OH)_2_D modulated glycolysis in cancer models *in vitro*. However, it is important to note that the extent and manner of this regulation may differ across various types. Furthermore, 1,25(OH)_2_D treatment reduced the protein levels of hexokinase 2, an enzyme that plays a crucial role in the initial stages of glycolysis, in MCF-7 breast cancer cells, but increased hexokinase 2 protein expression in MDA-MB-231 cells.^85^

Research findings indicate that 1,25(OH)_2_D reduces lipid accumulation in cancer cells, emphasizing the possible role of lipid metabolism in Vit D-induced anti-cancer effects.^86^, ^87^, ^88^ Additionally, 1,25(OH)_2_D treatment inhibited *de novo* fatty acid synthesis and lipid accumulation in metastatic breast cancer cells by suppressing the expression of the anaplerotic enzyme pyruvate carboxylase.

Vit D has the potential to indirectly influence cell development by disrupting the activity of growth hormones that facilitate cell proliferation or expedite cell differentiation.^87^ MCF-7 cell proliferation was inhibited by Vit D analogs in the presence of insulin-like growth factor 1 (IGF1), and this effect was accompanied by an increase in the release of insulin-like growth factor-binding protein 3 (IGFBP3). IGFBP3 has an affinity for IGF1 and IGF2, which suppresses their interaction with cell surface receptors, thereby diminishing their proliferative and anti-apoptotic effects.^89^

Furthermore, the development of new blood vessels necessary for the growth and propagation of solid tumors depends on the presence of vascular endothelial growth factor (VEGF).^89^ The Vit D analog calcitriol has been shown to inhibit the transcription and expression of HIF-1 and reduce the expression of VEGF. Calcitriol exerts anti-angiogenic effects via the nuclear factor kappa B (NF-κB) signaling pathway, involving the nuclear proteins FOXM1 and DKK4. Additionally, calcitriol suppresses the activity of IL-8, a significant factor involved in angiogenesis.

### Vitamin E

α-Tocopheryl succinate is widely recognized as a highly potent derivative of Vit E, exerting inhibitory effects against cellular growth and triggering apoptosis in several cancer cell lines.^90^ Importantly, the potential anti-cancer effects of α-tocopheryl succinate could be attributed to its capacity to hinder oxidative phosphorylation at the mitochondrial complexes I and II, thus promoting ROS production. ROS production triggers the activation of *NRF2-*dependent antioxidant genes, leading to potential therapeutic outcomes.^91^ However, the cytoprotective effects of α-tocopheryl succinate enhance the resistance of PC3 prostate cancer cells to oxidative damage caused by chemotherapeutic agents.^92^ α-Tocopheryl succinate treatment for a brief duration of 4 h decreased cell survival, increased ROS production in PC3 prostate cancer cells, and elevated the intracellular content of GSH.^92^

## Molecular interactions of vitamins on cancer cells

Vits A, B, C, and D have diverse molecular interactions with cancer cells that regulate intricate cellular processes in tumorigenesis, such as DNA damage, cell cycle arrest, and apoptosis induction.^93^ Although extensive research has been conducted on the anti-cancer effects of vitamins, there is limited understanding of the integrated biomolecular mechanisms of the interaction of these vitamins.

Notably, the activities of Vit A, particularly its active form RA, are mediated by its interaction with specialized nuclear receptors called RA receptors (RARs) and retinoid X receptors (RXRs). The binding of the RAR-RXR complex to distinct DNA sequences located in the promoter regions of target genes, referred to as RA response elements (RAREs), regulates Vit A and RA expression. The observed reaction has been researched in homeobox A1 (Hoxa1), RA receptor beta2 (RARβ2), and Cyp26A1 RAREs when F9 embryonal carcinoma cells are treated with RA.^94^ RA treatment induced the competitive inhibition of the suppressor of zest 12 (SUZ12), a protein that plays a vital role in polycomb repressive complex 2 (PRC2)-mediated gene silencing by facilitating trimethylation of the lysine 27 residue of histone H3 (H3K27me3). This trimethylation process is crucial for the regulation of tumorigenesis in various cancer types, including lung and colorectal cancer.^84^

Another vitamin involved in DNA binding and regulation is Vit D, whose active form, calcitriol, interacts with cancer cells through similar mechanisms by binding to VDR. VDR is a transcription factor that forms a complex with RXR upon ligand binding and binds to Vit D response elements (VDREs) in the DNA of target genes.^95^ Direct binding of VDR to the response element in genes that regulate cell growth can affect cell proliferation or major transcriptional regulators that are involved in cell signaling molecules engaged in the cell cycle, apoptosis, and differentiation.^95^

VDREs exert repressive activity downstream of the ER promoter regions in breast, prostate, and colon cancers.^96^ Additionally, RA and calcitriol can alter the expression of genes involved in DNA repair and genomic integrity.^97^ RA stimulates the production of enzymes involved in DNA repair pathways such as nucleotide and base excision repair. Moreover, these repair systems are critical for maintaining DNA integrity, reducing mutation accumulation, and ensuring precise and efficient DNA synthesis during cell division, all of which help prevent cancer development.^97^

Research findings indicate that vitamins can cause G1 phase arrest and prevent cell progression into the S phase through the upregulation of cyclin-dependent kinase (CDK) inhibitors (CDKIs), which are essential for cell cycle progression, thereby halting cancer cell growth.^98^, ^99^, ^100^ In acute promyelocytic leukemia cells, RAR and CDK-activating kinase interact, and CDK-activating kinase inhibition by all-trans RA (ATRA) results in hypophosphorylation of PML-RARα and myeloid differentiation. ATRA- and Vit D3-induced downregulation of CDK activity is frequently caused by an increase in the levels of p21- and p27-bound CDKs.^101^ Additionally, the CDK inhibitor p27/kip1 was modulated by ATRA-induced suppression of the growth of the ovarian cancer cell line CAOV3.^100^ Further studies have revealed that ATRA activates Rb protein and inactivates E2F1 to increase the levels of p16, p21, and p27 in human liver cell lines, which inhibits the growth of cancer cells.^100^^,^^102^

Calcitriol also increased cyclin D1 and E degradation by upregulating p21 and p27, resulting in G1/G0 arrest. Moreover, Vit D treatment decreased C-MYC expression and increased Rb protein levels in cell lines, both of which induced G1/G0 arrest.^103^ Furthermore, Vit C (ascorbic acid) regulates cell cycle and apoptosis via several mechanisms, most of which are related to its antioxidant characteristics and modulation of intracellular signaling pathways. Notably, the activities of Vit C are primarily achieved by ROS production within cells, which initiates DNA damage responses and activates checkpoint proteins, resulting in cell cycle arrest. Consistent with the above findings, IV administration of high-dose of Vit C as an adjuvant ameliorated the toxic side effects of chemotherapy.^102^

Among the hallmarks of cancer development, anti-apoptotic mechanisms are crucial for cancer development and progression. Vits C and D and RA have been shown to combat this process and promote apoptosis in cancer cells by activating several pathways, especially those that upregulate pro-apoptotic proteins (Bax and Bak) and downregulate anti-apoptotic proteins (B-cell lymphoma 2 [Bcl-2]), as shown in Figure 4. Importantly, the activation of caspases and subsequent apoptosis caused by an imbalance between pro- and anti-apoptotic proteins prevents cancer development and progression.^104^ For instance, treatment with methyl donors (Vit B9 and B12) increased apoptosis in the breast and lung cancer cell lines, including T47D, H1650, and A549, by increasing the expression of the pro-apoptotic proteins Bak and Bax and caspase-9 accumulation.^104^ Previous studies using high concentrations of Vit A derivatives, such as ATRA and its analog (13 cis RAs), have revealed similar outcomes in the hepatoma cell lines Hep3B and HepG2.^104^, ^105^, ^106^ Notably, superior results were observed in HepG2 cells treated with ATRA, as evidenced by enhanced apoptosis, B-cell lymphoma-extra large (Bcl-xL) downregulation, Bax upregulation, and cleavage of procaspases-3 and-8.^107^^,^^108^ In some drug-resistant breast cancer cell lines, Vit C induced apoptosis without altering p53 and exerted anti-proliferative effects. Additionally, Vit B2 increased the susceptibility of cancer cells to Vit C-induced cell death.^109^ Preliminary research indicates that Vit D directly causes apoptosis in prostate cancer cells by suppressing the anti-apoptotic gene *Bcl-2*.^110^ Recent studies revealed that the Vit D derivative calcitriol can directly affect the expression of genes implicated in apoptosis, such as tumor necrosis factor-related apoptosis-inducing ligand (TRAIL) and Fas ligand, thereby enhancing apoptotic cell death.^111^^,^^112^

Different molecular interactions between Vits A, B, C, and D and cancer cells affect cellular functions, such as cell division, proliferation, apoptosis, and DNA synthesis.^113^ Although these vitamins have potential as anti-cancer drugs, further studies are necessary to comprehensively elucidate the mechanisms of action of vitamins and identify the most effective therapeutic modalities.

## Effects of excess vitamins on cancer cells

Vitamins are vital nutrients that humans require in small quantities to maintain good health and proper biological functions. Vitamins play important roles in various processes, including supporting growth, boosting the immune system, promoting energy production, and ensuring the well-being of the skin, bones, and organs.^114^ Although vitamins are crucial for well-being, it is necessary to avoid excessive vitamin intake, especially when undergoing cancer treatment, as it can lead to a condition known as hypervitaminosis. Hypervitaminosis can have varying symptoms depending on the specific vitamin involved; however, common symptoms include nausea, vomiting, diarrhea, fatigue, dizziness, and organ damage in severe cases.^101^ Typically, excessive intake of highly fortified foods is the main cause of toxicity and hypervitaminosis, resulting in the accumulation of fat-soluble vitamins in the liver.^115^ Hypervitaminosis is related to fat-soluble vitamin groups, such as Vits A, D, E, or K, which tend to have a more pronounced impact than water-soluble vitamins, such as B6, and B12. Moreover, fat-soluble vitamins can accumulate in body tissues and cause severe intoxication, as they are not eliminated through urine.^101^^,^^115^

Epidemiological studies suggest that a diet rich in fruits and vegetables containing carotenoids, along with high blood levels of Vit E (αT) and β-carotene may reduce the risk of lung cancer.^101^^,^^114^^,^^115^ However, a study involving male smokers showed that αT and β-carotene supplementation for several years did not reduce lung cancer incidence.^101^ Another study indicated that Vit E increases the activity of carcinogen-bioactivating enzymes and promotes DNA damage and cell transformation in prostate epithelial cells, potentially increasing the risk of prostate cancer.^10^

### Vitamins in clinical trials for cancer treatment

Clinical trials have been performed to investigate the therapeutic effects of both water- and fat-soluble vitamins in various health conditions such as cancer, anemia, asthma, diabetes mellitus, and pediatric Crohn's disease.^11^ Clinical trials examining the effects of Vit A (retinoids), C (ascorbic acid), D, and E (tocopherols and tocotrienols) in cancers are either in the active, completed or participant recruitment phases, with only a few terminated trials with results. Moreover, several vitamins are being tested as co-drugs along with potential therapeutics, suggesting that they may enhance the activity of existing or potential drugs.

Currently, the National Library of Medicine lists >400 active clinical studies on vitamins and their role in combating cancer. Considering the substantial number of clinical trials, it is evident that several vitamins have demonstrated promising results against various types of cancers both *in vitro* and *in vivo*.^116^ Overall, these preliminary findings, along with ongoing clinical investigations, highlight the considerable potential of vitamins in the treatment of cancers and other diseases. Presently, approximately 40 clinical trials are ongoing to investigate the role of Vit D in cancer.^116^ Collectively, the results of these clinical trials indicate that vitamins may play vital roles in cancer biology and overall human health.

The drug-like properties of these vitamins are listed in Table 1. Lipinski's Rule of Five, Verber, Ghose, and Egan's rules are important guidelines for drug discovery. These rules are essential guidelines for drug discovery to evaluate the drug-like characteristics of chemical compounds and aid researchers in selecting potential candidates with high oral bioavailability and therapeutic efficacy. As shown in Table 1, all vitamins complied with Lipinski's Rule of Five, which is one of the most important properties of potential orally available drugs. However, Vits C, D, E, and K violated at least one of Ghose's, Verber's, or Egan's rules. Notably, their bioavailability properties suggest that they can be considered co-drugs.

**Table 1:** The drug-likeness properties of vitamins A, C, D, E, and K along with their respective PubChem compound identifier (CID)^12^.

Vitamins	PubChem CID	Drug-like properties				
		Lipinski	Ghose	Veber	Egan	Bioavailability
Vitamin D (cholecalciferol)	5280795	Yes; 1 violation: MLOGP > 4.15	No; 2 violations: WLOGP > 5.6, #atoms > 70	Yes	No; 1 violation: WLOGP > 5.88	0.55
Vitamin C (ascorbic acid)	54670067	Yes; 0 violation	No; 2 violations: WLOGP < −0.4, MR < 40	Yes	Yes	0.56
Vitamin E (α-tocopherol)	14985	Yes; 1 violation: MLOGP > 4.15	No; 3 violations: WLOGP > 5.6, MR > 130, #atoms > 70	No; 1 violation: rotors > 10	No; 1 violation: WLOGP > 5.88	0.55
Vitamin A (retinol)	445354	Yes; 1 violation: MLOGP > 4.15	Yes	Yes	Yes	0.55
Vitamin K	5280483	Yes; 1 violation: MLOGP > 4.15	No; 3 violations: WLOGP > 5.6, MR > 130, #atoms > 70	No; 1 violation: rotors > 10	No; 1 violation: WLOGP > 5.88	0.55

## Perspective and future scope

Based on the limited available molecular and biochemical data, it can be inferred that several vitamins may influence multidrug cancer resistance through various mechanisms, including through the regulation EMT and suppression of cancer stem cells. Additionally, the potential effects of vitamins on the regulation of oncogenes through the modulation of microRNA (miRNA) expression warrants further research. Overall, vitamin administration may be a potential therapeutic approach for targeting tumors with a multidrug-resistant phenotype. Additionally, research evidence indicates that vitamins exhibit potent cancer-selective cytotoxicity, sensitize cancer cells to therapy, and reduce toxicity when administered intravenously and at high doses. Notably, the administration of high doses of vitamins can improve the therapeutic outcomes of radiotherapy, chemotherapy, and targeted therapies. Moreover, the augmented therapeutic effects of immune checkpoint inhibitors and high-dose vitamins may prove advantageous for several cancer patients. However, inadequate accrual continues to impede further clinical investigation, primarily because the drug combination under scrutiny is no longer the prevailing standard of care. Further clinical investigations that combine high-dose vitamins with immunotherapy may address this issue. Considering that high-dose vitamins may be used in the treatment of patients with poor prognosis and limited therapeutic alternatives, further clinical investigations of cancer treatment modalities are necessary.

### Recent and important achievements

Clinical trials are currently ongoing to investigate the possible advantages of incorporating Vit D supplements into existing therapies for patients with cancer. The phase 3 SOLARIS trial investigated the combined effect of high-dose Vit D3, chemotherapy, and bevacizumab on the progression-free survival of patients with advanced or metastatic colorectal cancer.^117^ Currently, researchers are investigating the anti-cancer properties of Vit D using models with structures similar to that of Vit D, without the harmful consequences of high concentrations.^118^^,^^119^ Ongoing clinical trials are evaluating the efficacy of Vit D and its analog paricalcitol, either alone or in conjunction with other therapies such as immunotherapy and chemotherapy, in patients with pancreatic cancer.^119^

A previous study reported varying occurrences of Vit D insufficiency among racial and ethnic groups and its potential contribution to certain cancer inequalities.^120^ Based on National Health and Nutrition Examination Survey (NHANES) data from 2011 to 2014, the prevalence of Vit D deficiency, defined as a serum 25-hydroxy Vit D concentration <30 nmol/L, was 18% in non-hispanic black adults, 2% in non-Hispanic white adults, 8% in non-Hispanic Asian adults, and 6% in Hispanic adults. Additionally, black individuals are less inclined to use Vit D pills than white individuals.^121^

Currently, ongoing observational studies are examining how Vit D levels and supplementation affect cancer risk through biological processes.^122^ Additionally, studies are investigating whether the positive effects of Vit D on cancers are limited to individuals with specific genetic variations in the genes responsible for Vit D metabolism or transportation.^123^ An assessment of a US population revealed enhanced cancer survival rates, mainly among women and men with a particular variant of the Vit D-binding protein, known as GC.^124^

Multiple randomized controlled studies have investigated the effect of Vit D supplementation on the risk of cancer-related mortality, with different outcomes.^125^^,^^126^ In the Vitamins and Lifestyle (VITAL) study, Vit D did not decrease the overall cancer deaths, although a decrease in mortality was observed when fatalities in the initial years of follow-up were removed.^127^ The D-Health experiment involving Australian individuals aged ≥60 years, found that a monthly dose of 60,000 IU of Vit D for 5 years did not decrease cancer mortality. A meta-analysis of 10 randomized controlled trials, including VITAL trial, revealed that Vit D supplementation caused a modest 13% decrease in cancer mortality over a follow-up period of 3–10 years.^127^ A meta-analysis of 21 randomized studies concluded that there is no evidence linking Vit D supplementation to decreased mortality from all-cause or cardiovascular disease.^128^ Notably, several patients in these trials had sufficient blood Vit D levels to ensure good general health.^129^ Additionally, the effect of Vit D supplementation on cancer-related mortality may be more pronounced in patients with low Vit D levels.^129^

Vitamins play a crucial role in various fundamental biological processes, such as DNA replication and transcription.^130^ Importantly, vitamins play a significant role in cancer development and prevention. Vitamins have shown promise in cancer diagnostics; however, their potential as biomarkers remains largely unknown. Research findings indicate that high levels of vitamins in the blood are associated with both decreased and increased cancer risks.^130^, ^131^, ^132^ Therefore, additional data are required to evaluate the efficacy of vitamins as biomarkers and assess their applicability to specific cancer types. Additionally, it is crucial to investigate whether increased levels of vitamins in the blood and/or tissues constitute a significant factor in the development of specific types of cancer or whether they are a result of an existing tumor. Measuring vitamin levels in the bloodstream is a non-invasive and widely applicable analytical procedure with great potential for enhancing diagnosis and prognosis.^133^

However, existing research has limitations that must be recognized. For example, the therapeutic effects and mechanisms of vitamins in tumors may be affected by variations in dosage methods in human and animal research, which may affect result translation.^134^ Animal models provide initial insights, but may not accurately mimic human metabolic processes, resulting in variations in human bioavailability and effectiveness.^135^ Considering that *in vitro* findings suggest that Vit A and E may be toxic to cancer cells, their use may not be beneficial *in vivo* in patients with cancer.^136^ Integrating preclinical and clinical research emphasizes the necessity for specific techniques tailored to the type of cancer, its stage, and patient features. Additionally, there are important gaps in our knowledge, such as the optimal effective and safe doses of vitamins, the underlying mechanisms of vitamins, clinical applications of vitamins, personalized treatment strategies, and long-term effects of vitamin supplementation in patients with cancer.^137^ Therefore, it is essential to conduct thorough interdisciplinary research to address these issues and facilitate the application of vitamins in the field of oncology.

Although there were variations in results across studies, this review emphasizes several common patterns. Most cases have shown an inverse correlation between Vit D levels and cancer risk. Low vitamin levels serve as cofactors in oncogenesis. This tendency aligns with the concept of hidden hunger, in which a diet lacking micronutrients leads to an imbalance in cellular biochemistry, which increases the risk of cancer development.^138^ The correlation between Vit D levels and cancer risk varies, depending on the organ involved. Vit B complex is deficient in almost all cancer types except for lymphomas, and Vit C levels are low in prostate and gastric cancer, but high in colon cancer.^139^

Notably, the cells of the body have strong mechanisms to maintain vitamin levels and repair bimolecular damage, making it improbable for temporary imbalances in these vitamins to cause cancer. However, long-term imbalances in vitamins can alter the cellular environment. Simply taking multivitamin/mineral supplements does not ensure the alleviation of hidden hunger, although they can help address temporary deficiencies in micronutrients.^135^, ^136^, ^137^ This limitation implies that measuring the levels during a specific period may not provide significant diagnostic or prognostic insights. Longitudinal analysis of vitamin concentration can offer insights into the potential risk of DNA damage caused by oxidative stress, structural weakness, and epigenetic abnormalities.^140^ Individuals may measure the levels of vitamins in their blood over time using commercial instruments similar to cholesterol or glucometers, which may help determine whether specific vitamins or minerals are below normal ideal levels. Additionally, the levels of vitamins may be associated with the risk of DNA damage and may be used to identify individuals with an increased risk of cancer.

## Conclusions

Vitamins are commonly recognized as essential nutrients that serve as precursors to hormones and play a pivotal role in regulating several physiological processes. In addition to their traditional functions in bone metabolism, recent epidemiological, preclinical, and cellular studies suggest that vitamins may play important roles in the prevention and management of various diseases, including cancer. Additionally, vitamins possess several anti-cancer properties and exert varying effects on cancer development and progression. Overall, research evidence indicates that vitamin metabolism and functions are integrated in various cancer types, resulting in the activation of the anticancer properties of vitamins. This phenomenon is believed to play an important role in the onset and treatment of cancer. Therefore, understanding the effect of alterations in the metabolism and function of vitamins in cancer is crucial for effective treatment.

A comprehensive analysis of the scientific literature on the effect of vitamins on cancer risk indicated that vitamins play a crucial role in cancer therapy, despite the presence of inconsistent findings. Vitamins exert beneficial effects on cancer through their anti-proliferative and apoptotic properties. Importantly, combined treatment with vitamins and various drugs have been shown to induce these effects and enhance the efficacy of medical interventions. Additionally, studies involving animal and cell culture models have provided significant evidence of the crucial role of vitamins-associated anti-inflammatory properties in cancer treatment.

Recently, the role of vitamins in cancer prevention and treatment has attracted considerable research attention. Vitamins play important roles in preventing cancer by affecting immunological functions, antioxidant defenses, inflammation, epigenetic regulation, cell differentiation, proliferation, apoptosis, oxidative stress, and DNA damage. Vit A plays a regulatory role in cell proliferation and differentiation and affects several signaling pathways associated with cancer. Vit C acts as an antioxidant, decreases oxidative stress, and boosts immune responses to combat cancer. IV administration of high-dose of Vit C can specifically target cancer cells. Additionally, the antioxidant properties of Vit E protect cell membranes from damage, and some types of Vit E have anti-inflammatory effects. Vit D is associated with a decreased risk of certain malignancies, such as colorectal cancer, owing to its involvement in cell development and immunological function. Vits A, C, D, and E supplementation combined with chemotherapy and radiotherapy provides a tailored approach for cancer treatment. Overall, these vitamins boost the immune system, reduce oxidative stress, prevent the formation of new blood vessels, and trigger cell death. However, further studies are necessary to fully elucidate the potential of these therapies in cancer.

## Authors contribution

Abdullahi T. Aborode: conceptualization; Abdullahi T. Aborode, Isreal A. Onifade, Mercy M. Olorunshola, Gladys O. Adenikinju, Ibude J. Aruorivwooghene, Adeboboye C. Femi, Abosede Salami, Ogundepo D. Moyinoluwa, and Godfred Y. Scott: formal analysis; Ruth A. Ogbonna, Emmanuel A. Fajemisin, and Omama Ehtasham: clinical assessment and data record. All the authors contribute to the manuscript preparation. All the authors interpreted the data, critically reviewed the content, and significantly contributed to the writing of the manuscript. All the authors have read and approved the final version of the manuscript.

## Ethics statement

None.

## Declaration of generative AI and AI-assisted technologies in the writing process

The authors declare that generative artificial intelligence (AI) and AI-assisted technologies were not used in the writing process or any other process during the preparation of this manuscript.

## Funding

This research did not receive any specific grant from funding agencies in the public, commercial, or not-for-profit sectors.

## Data availability statement

The datasets used in the current study are available from the corresponding author on reasonable request.

## Conflict of interest

The authors declare that they have no known competing financial interests or personal relationships that could have appeared to influence the work reported in this paper.
